# Disparities in Survival After In-Hospital Cardiac Arrest by Time of Day and Day of Week: A Single-Center Cohort Study

**DOI:** 10.3390/jcm15030987

**Published:** 2026-01-26

**Authors:** Maria Aggou, Barbara Fyntanidou, Marios G. Bantidos, Andreas S. Papazoglou, Athina Nasoufidou, Aikaterini Apostolopoulou, Christos Kofos, Alexandra Arvanitaki, Nikolaos Vasileiadis, Dimitrios Vasilakos, Haralampos Karvounis, Konstantinos Fortounis, Eleni Argyriadou, Efstratios Karagiannidis, Vasilios Grosomanidis

**Affiliations:** 1 Department of Anesthesiology, AHEPA University Hospital, 54636 Thessaloniki, Greece; mariaangou@auth.gr (M.A.); dvasilak@auth.gr (D.V.); argiriadou@auth.gr (E.A.); vgrosoma@auth.gr (V.G.); 2 Department of Emergency Medicine, AHEPA University Hospital, 54636 Thessaloniki, Greece; fyntanidou@auth.gr (B.F.); mpantidos@auth.gr (M.G.B.); apostoo@auth.gr (A.A.); ekaragij@auth.gr (E.K.); 3 Second Department of Cardiology, Hippokration General Hospital, 54642 Thessaloniki, Greece; athinaso@auth.gr (A.N.); ckofos@auth.gr (C.K.); 4 Department of Cardiology, Athens Naval Hospital, 11521 Athens, Greece; aspapazo@auth.gr; 5 Amedes Medizinische Dienstleistungen GmbH, 37081 Göttingen, Germany; nikolaos.vasileiadis@hotmail.com; 6 First Department of Cardiology, AHEPA University Hospital, 54636 Thessaloniki, Greece; hkarvounis@auth.gr; 7 Department of Surgery, Papageorgiou General Hospital, 56429 Thessaloniki, Greece; kostfort@yahoo.gr

**Keywords:** in-hospital cardiac arrest, mortality, off-hours, resuscitation, shift, weekend

## Abstract

**Background:** In-hospital cardiac arrest (IHCA) constitutes a high-impact clinical event, associated with substantial mortality, frequent neurological and functional impairment. There is a pressing need for primary IHCA studies that evaluate risk predictors, given the inherent challenges of IHCA data collection, previously unharmonized reporting frameworks, and the predominant focus of prior investigations on other domains. Among potential contributors, the “off-hours effect” has consistently been linked to poorer IHCA outcomes. Accordingly, we sought to examine whether in-hospital mortality after IHCA varies according to the time and day of occurrence within a tertiary academic center in Northern Greece. **Methods:** We conducted a single-center observational cohort study using a prospectively maintained in-hospital resuscitation registry at AHEPA University General Hospital, Thessaloniki. All adults with an index IHCA between 2017 and 2019 were included, and definitions followed Utstein-style recommendations. **Results:** Multivariable logistic regression adjusted for organizational, patient, and process-of-care factors demonstrated that afternoon/night arrests, weekend arrests, heart failure comorbidity, and need for mechanical ventilation were independent predictors of higher in-hospital mortality. Conversely, arrhythmia as the cause of IHCA and arrests occurring in the intensive care unit or operating room were associated with improved survival. Subgroup analyses confirmed consistent off-hours differences, with weekend events showing reduced 30-day and 6-month survival and worse functional status at discharge. Afternoon/night arrests were more frequent, characterized by longer response intervals and lower survival at both time points. **Conclusions:** Organizational factors during nights and weekends, rather than patient case mix, drive poorer IHCA outcomes, underscoring the need for targeted system-level improvements.

## 1. Introduction

### 1.1. Background

In-hospital cardiac arrest (IHCA) is defined in the Utstein resuscitation registry reporting template as the delivery of chest compressions and/or defibrillation to inpatients [1]. IHCA represents an often-neglected serious event for hospitalized patients, related to substantial mortality and functional impairment.

### 1.2. Epidemiology and Outcomes

The incidence of IHCA differs between countries, ranging from 1.6 to 8.3 per 1000 hospital admissions [2,3,4,5,6]. Outcomes do not only include survival but also long-term neurological status and the requirement of physiotherapy, nursing, occupational therapy, and additional medical care [7]. Outcomes after IHCA are closely related to early recognition, rapid assembly of dedicated IHCA teams, high-quality resuscitation, and ongoing performance evaluation [8]. Data from a large U.S.A. registry, including 84,625 patients, demonstrated that survival and neurological outcomes after IHCA have been improved in general over the last decade [9].

### 1.3. Challenges in Obtaining Clinical Data

IHCA has not received as much attention as out-of-hospital cardiac arrest, as seen in a meta-analysis of 92 trials on cardiac arrest, of which only 4% of the studies reported exclusively on IHCA [10]. This negligence can be attributed to two main reasons: the difficulty in collecting real-time clinical data in variable settings and the inconsistency in reporting outcomes before the Utstein definitions. Regarding the pre-Utstein era, studies used different variable sets, definitions and measures for outcomes [e.g., different definitions for return of spontaneous circulation (ROSC) or time intervals], making comparisons almost impossible [11].

### 1.4. Suboptimal Outcomes—Time and Day of the Week

Outcomes after IHCA are multifactorial, and prior evidence has recognized determinants that negatively contribute to survival and recovery. More specifically, prior literature suggests worse outcomes when IHCA occurs during nighttime or weekends, especially on wards, reflecting weak points in the IHCA management algorithm. These include lower staffing levels during off-hours, reduced monitoring, slower escalation of care, but also institution-specific factors [12,13].

### 1.5. Knowledge Gap and Study Objective

Despite existing evidence on mortality after IHCA, there are gaps regarding how temporal factors, like the exact time and day of the week the event occurs, may contribute to poor outcomes and undermine the patients’ recovery. Moreover, factors like the bedside staffing during off-hours, monitoring intensity, and team responsiveness remain insufficiently characterized in studies across Europe. To address these gaps, the present study aims to evaluate whether IHCA mortality varies by timing of arrest within a tertiary academic center, using comprehensive adjustment for patient, organizational, and resuscitation-related factors. This approach seeks to clarify the mechanisms underlying off-hours disparities and inform targeted strategies to improve IHCA outcomes across all hospital periods.

## 2. Materials and Methods

### 2.1. Study Design, Setting and Ethical Approval

This study was conducted as a single-center observational cohort study, based on a prospectively collected institutional registry of IHCA events at AHEPA University General Hospital, Thessaloniki, Greece, covering the period 2017–2019. This period was selected because it corresponds to a phase of stable, complete Utstein-style registry capture at our institution and was intentionally restricted to the pre–COVID-19 era to avoid pandemic-related disruptions in case-mix, staffing, and intensive care unit (ICU) capacity. The study protocol received approval by the Directory Board and the Scientific Council of AHEPA University General Hospital. The requirement for individual informed consent was waived due to the retrospective analysis of the prospectively assessed entries. Data handling complied with institutional policies and applicable data-protection legislation (European Union General Data Protection Regulation, EU GDPR).

### 2.2. Data Source and Population

We used a prospectively maintained in-hospital resuscitation registry to identify all eligible cases. Inclusion criteria were adult patients ≥ 18 years who experienced a documented IHCA. Patients with out-of-hospital cardiac arrest and those with a preexisting order for do-not-attempt-resuscitation (DNAR) were excluded from the study.

### 2.3. Definitions and Variables

Data collection and definitions followed the harmonized Utstein-style definitions from the 2004 and 2019 guideline frameworks to ensure standardized reporting [1]. The primary exposure variables were the timing of the arrest, defined by time of day (morning, 07:00–14:59 vs. afternoon/night, 15:00–06:59) and by day of week, defined using the calendar day of arrest as weekday (Monday 00:00 to Friday 23:59) or weekend (Saturday 00:00 to Sunday 23:59). These cutoffs were selected a priori to reflect the local shift transition from routine in-house staffing to greater reliance on on-call coverage. Although alternative categorizations are also used in the IHCA literature (e.g., day/evening/night or overnight definitions), we used a binary split aligned with local operational transitions and to ensure adequate subgroup sizes. To account for potential confounding factors, we incorporated a broad mix of covariates:

(i) Organizational factors included the location of the event within the hospital [wards, ICU/Operating Room (OR)] and the level of staffing (nurses and physicians) at the exact time of the event.

(ii) Patient-related covariates, accounting for demographic characteristics, comorbidities, and the initial admission diagnosis. Documented comorbidities included coronary artery disease (CAD), chronic kidney disease (CKD), cancer, diabetes mellitus (DM), heart failure (HF), valvular heart disease (VHD), previous stroke, hypertension (HTN), pulmonary pathology, endocarditis, and mental illness. Initial admission diagnoses included all the aforementioned comorbidities, as well as coma, cardiac arrhythmia, aortic dissection, gastrointestinal pathology, hematological disorders, syncope, and trauma.

(iii) Process of care factors were also recorded, taking into consideration the resuscitation team response times, the total duration of the cardiopulmonary resuscitation, and the timing of airway intervention by specialists.

This comprehensive framework allows the thorough evaluation of how the temporal elements influence IHCA outcomes and gives answers to our study’s objective.

### 2.4. Outcomes

The primary outcome of the study was the in-hospital mortality. As secondary outcomes, mortality was also evaluated at prespecified time frames, including the 30-day and 6-month survival. Functional status at discharge was also assessed, using the Cerebral Performance Category (CPC) scale, a standardized measure of neurological outcome after cardiac arrest [14]. CPC was evaluated among patients discharged alive with available documentation.

### 2.5. Statistical Analysis

Continuous variables were assessed for normality; normally distributed variables were summarized as mean (standard deviation, SD), while non-normally distributed variables were reported as median (interquartile range, IQR). For the evaluation of the primary outcomes, we used multivariable logistic regression. Results were expressed as adjusted odds ratios (aORs) accompanied by 95% confidence intervals (CI) and a two-sided significance threshold of α = 0.05. The primary exposures were time of day (morning vs. afternoon/night) and day of week (weekend vs. weekday). To account for potential confounding, we used an a priori adjustment set including clinically and organizationally relevant variables, along with key process-of-care time intervals.

To maximize sample retention for process variables, we applied simple single-value imputation for predictors only (median for continuous, mode for categorical), leaving outcomes unaltered; predictors with no variation after imputation were excluded. Model complexity was intentionally limited and assessed relative to the number of outcome events to reduce the risk of overfitting. The model performance was evaluated using multiple diagnostic tools: multicollinearity was inspected using variance inflation factors (VIFs), with values < 5 considered acceptable; calibration was examined using the Hosmer–Lemeshow goodness-of-fit test; and discrimination was quantified by the area under the receiver operating characteristic curve. In addition to the initial analysis, prespecified subgroup comparisons were conducted to further describe outcome differences across the temporal exposure categories.

## 3. Results

### 3.1. Cohort Description

A total of 826 IHCA events were eligible for inclusion in the study, representing 826 unique patients. The median age was 73 years [IQR: 61–80], and the majority were male patients (65%). The most common comorbidities appeared to be chronic CAD (25%), HF (11%), and cancer (7%).

### 3.2. Primary Model Performance

The multivariable logistic regression model demonstrated good performance across the multiple prespecified metrics. Calibration of the model was adequate, with a Hosmer–Lemeshow goodness-of-fit test yielding a *p*-value of 0.423, reflecting agreement between observed outcomes and predictions. Model discrimination was satisfactory, with an area under the receiver operating characteristic curve of 0.744, indicating a reasonable ability to differentiate survivors from non-survivors after IHCA. Multicollinearity assessment has shown low interdependence among predictor variables, with all VIFs ≤ 3.2, reinforcing the stability and reliability of the estimated effects.

### 3.3. Predictors of In-Hospital Mortality

Multivariable logistic regression revealed multiple factors independently related to increased in-hospital mortality after IHCA. IHCAs during the afternoon or night were associated with a 1.75-fold increased risk of death (aOR = 1.75, 95% CI 1.17–2.61; *p* = 0.007), while events occurring during weekends demonstrated an even higher risk (aOR = 2.03, 95% CI 1.21–3.54; *p* = 0.009). Regarding patient-related factors, preexisting HF increased notably the mortality risk (aOR = 2.57, 95% CI 1.38–5.19; *p* = 0.005), as well as the need for mechanical ventilation at the time of the event (aOR = 2.61, 95% CI 1.60–4.23; *p* < 0.001). On the contrary, cardiac arrhythmia as a cause of IHCA was related to lower mortality (aOR = 0.29, 95% CI 0.18–0.47; *p* < 0.001), as was the location of the IHCA when that was the ICU or the OR (aOR = 0.17, 95% CI 0.07–0.40; *p* < 0.001). The full multivariable model is presented in Table 1.

### 3.4. Subgroup: Weekend Versus Weekdays

Subgroup analyses of weekend vs. weekday IHCAs showed that patients suffering from IHCA on weekends compared to weekdays had more frequent acute myocardial infarction as their initial admission diagnosis (21% vs. 14%, *p* = 0.029) and, during resuscitation, showed higher rates of nurse participation (99% vs. 96%, *p* = 0.018) but lower rates of physician presence (27% vs. 37%, *p* = 0.007). Furthermore, weekend IHCAs were also associated with lower survival at 30 days (10% vs. 19%, *p* = 0.003) and 6 months (8.7% vs. 17%, *p* = 0.004), as well as lower functional status at discharge (*p* = 0.035). No statistically significant differences were noted between groups in other baseline comorbidities, initial rhythm categories, or process-of-care intervals. Table 2 emphasizes patient-, event-, and system-level variables with plausible relevance to the off-hours effect; complete variable-level comparisons are provided in Supplementary Table S1.

### 3.5. Subgroup: Morning Versus Afternoon/Night

Moreover, we analyzed the events based on the time of day. Compared with morning arrests, those occurring in the afternoon/night were more frequent (505 vs. 321) and had longer team arrival (3 vs. 2 min, *p* = 0.037), longer cardiopulmonary resuscitation duration (20 vs. 15 min, *p* = 0.043), longer time to airway establishment (*p* = 0.040), fewer operating room arrests (1.6% vs. 7.2%, *p* < 0.001), lower prevalence of ventricular fibrillation as the initial rhythm (17% vs. 23%, *p* = 0.039), and lower survival both at 30 days (14% vs. 22%, *p* = 0.002) and 6 months (12% vs. 20%, *p* = 0.001), with no difference in functional status at discharge (*p* = 0.20). Table 3 emphasizes patient-, event-, and system-level variables with plausible relevance to the off-hours effect; complete variable-level comparisons are provided in Supplementary Table S2.

## 4. Discussion

### 4.1. Principal Findings

The principal finding of our study is that off-hours survival disadvantage persists, even after adjusting for multiple confounding factors after IHCA. The disadvantage appears to reflect case mix (fewer initial shockable rhythms) and care context [fewer arrests in advanced environments (ICU/OR) and lower physician presence on weekends]. Non-shockable rhythms are typically linked to late recognition, an aspect related to ward rounds compared to ICU [15]. Standard response intervals were broadly similar on weekdays vs. weekends, but not uniformly by shift (longer team arrival in the afternoon/night). These discrepancies reflect the negative impact of non-routine hours, like short staffing, no senior physician coverage, and disturbed overall workflow [16]. Importantly, preexisting monitoring percentages did not differ significantly across periods, indicating that-in addition to any constraints in monitoring equipment coverage-the decisive factors are likely at-the-bedside capability and escalation pathways at the time of arrest. Conversely, the protective association of ICU/OR plausibly reflects immediate defibrillation access, airway/ventilation expertise, and teams practiced in resuscitation workflows [17].

### 4.2. Comparison with the Existing Literature

The results of our study align with prior multicenter evidence demonstrating persistently lower survival from IHCA during nights and weekends, even after adjustment for patient and event characteristics.

Peberdy et al. analyzed 86,748 adult IHCA and found that survival rates were significantly worse during nighttime compared to day/evening hours, with lower ROSC rates, 24 h survival, and poorer neurological status. Also, asystole was more often the first diagnosed rhythm during nighttime arrests, and ventricular fibrillation was less frequent [18].

A large U.K. study based on two registries demonstrated diurnal variation in IHCA, with poorer outcomes overnight (20:00–8:00), with a marked peak at 6:00. On the contrary, no diurnal differences were seen among patients admitted to the ICU, suggesting that patient elements and the care system drive these results, rather than the circadian cycle [19,20].

In another UK study, ROSC > 20 min and survival since discharge were significantly decreased during off-hours, especially for non-shockable rhythms [21]. A U.S. study with 151,071 patients from 470 hospitals investigated the changes in survival between on-hours and off-hours at two time points. The authors found that overall survival has been improved over a 15-year time, but off-hours arrests continue to demonstrate lower rates. Remarkably, off-hours arrests accounted for 52% of total events [22].

Another study from a Swedish cardiopulmonary resuscitation registry categorized IHCA based on day (07:00–15:00), evening (15:00–21:00), and night (21:00–07:00 weekdays and all weekend hours). Survival in 30 days and ROSC rates were higher in the daytime (36.8% and 67.9%) and decreased progressively in the evening (32.0% and 66.3%) and nighttime (26.2% and 60.2%). Smaller hospitals and non-academic institutions demonstrated even more pronounced differences between day and night survival rates [23].

In a South Korean study, the so-called “weekend effect” was diminished one year after its recognition by employing more staff and improving workflow procedures [24]. A U.S.A. study reported that when the “Code Blue Team” was utilized in their center, survival rates did not vary between different times of the day and weekdays [25]. Finally, a retrospective study from Singapore found no association between the timing of IHCA and the rate of ROSC or 90-day survival [26]. The limited number of studies reporting no weekend effect in post-IHCA outcomes may be attributable to publication bias.

### 4.3. Implications for Practice

There are multiple modifications that can be made to improve outcomes after IHCA at both institutional and staff level. The initiation of the resuscitation protocol is the first and most crucial step. Second, the absolute survival gaps observed during off-hours (weekend vs. weekday and afternoon/night vs. morning) were clinically meaningful (approximately 8–9 percentage points for both 30-day and 6-month survival), suggesting that improving off-hours staffing for both nurses and physicians could help mitigate these. Given that a large proportion of IHCAs occurred during afternoon/night hours in our cohort (approximately twice as many as during morning hours), prioritizing staffing balance and response pathways during these periods may be a reasonable starting point with the potential for substantial hospital-level gains and may support targeted resource allocation. Rapid team response and streamlined communication with the ICU for escalation may further improve time-sensitive interventions and outcomes. In parallel, a standardized resuscitation team composition with access to the necessary expertise, combined with regular training that emphasizes both technical performance and non-technical skills (teamwork and communication), may improve performance and reduce individual-level variability [17].

### 4.4. Limitations and Strengths of the Study

Limitations of our study include the single-center design, which may limit the applicability of the findings to other hospital settings with different staffing rules, patient populations, or resuscitation protocols. Second, the observational design of our study precludes causal inference regarding the relationship between timing of arrest, patient or organizational factors, and outcomes. Although multivariable adjustment was performed, residual unmeasured confounding (e.g., institutional policies or variations in performance of teams/individuals) cannot be excluded. In addition, the registry captured whether a physician was present during resuscitation but did not systematically record physician seniority (resident vs. specialist) or the immediate availability of specific specialists (e.g., intensivist/cardiologist) at the case level; therefore, we could not directly examine whether these factors mediated the observed off-hours associations. We also could not fully account for DNAR/DNACPR decisions instituted during hospitalization, which may have differed across time periods and influenced outcomes. Functional status was assessed only at the time of hospital discharge, which may not reflect long-term neurological outcomes and physical recovery. Lastly, the cohort ends in 2019 due to the period of complete registry coverage and to avoid COVID-19–era system disruptions; therefore, practice changes after 2019 are not captured and should be considered when extrapolating these results to later periods.

Despite these limitations, several strengths can be recognized in our study. First, data collection, definitions, and reporting were based on harmonized Utstein-style definitions, incorporating both the 2004 and 2019 templates. This process verifies the consistency in the definition of IHCA events, measured time intervals, and outcomes for further comparisons to be reliable and consistent with the contemporary literature. Second, our statistical analysis incorporated a large list of covariates to allow adjustments of patient-level characteristics, organizational factors, and process-of-care measures. Minimizing confounding factors was of great importance to lead to a better understanding of the determinants of IHCA outcomes beyond standard known associations. Third, the study utilized a prospectively maintained registry in order to reduce the risk of reporting bias and amplify the accuracy and the completeness of the included data. Prospective data allow more precise timing and documentation, which further strengthen the reliability of the study. Finally, the single-center design within a tertiary academic hospital ensured consistent protocols and resuscitation practices, which would not be achieved in a multi-center study.

## 5. Conclusions

In summary, IHCA during off-hours, including nighttime and weekends, is associated with significantly lower survival and worse medium-term outcomes. Contributing factors include patient mix and hospital-level conditions that vary during these time periods. Enhancing off-hours staffing, resulting in early cardiac arrest recognition and rapid initiation of the resuscitation protocol, could improve IHCA outcomes significantly.

## Figures and Tables

**Table 1:** Multivariable logistic regression model for in-hospital mortality after in-hospital cardiac arrest.

Predictor	aOR	95% CI	*p*-Value
Time of day (afternoon/night vs. morning)	1.75	1.17–2.61	0.007
Day of week (weekend vs. weekday)	2.03	1.21–3.54	0.009
ARDS as direct cause	1.16	0.69–1.96	0.6
All ALS procedures present	2.28	0.91–6.30	0.093
Arrhythmia as direct cause	0.29	0.18–0.47	<0.001
Cardiac arrest in advanced department	0.17	0.06–0.40	<0.001
Cardiac arrest determination	1.07	0.57–2.02	0.8
Heart failure comorbidity	2.57	1.38–5.19	0.005
Mechanical ventilation present	2.61	1.60–4.23	<0.001
Nurse starting CPR	0.97	0.56–1.71	>0.9
Physician present during resuscitation	1.19	0.70–2.01	0.5
Time to CPR start	1.44	0.90–2.41	0.14
Time to resuscitation team arrival	1.02	0.75–1.40	0.9
Time to resuscitation team call	0.71	0.37–1.38	0.3
Time to airway establishment	1.16	0.93–1.46	0.2
Time to first adrenaline dose	1.00	0.85–1.20	>0.9
Time to first defibrillation	0.84	0.57–1.23	0.4

Adjusted odds ratios for time-to variables are reported per 1 min increase.

**Table 2:** Comparisons of patient-, event-, and system-level characteristics between weekday and weekend in-hospital cardiac arrests.

Variable	Weekend (n = 200)	Weekday (n = 626)	*p*-Value	
**Demographics**				
Age [median (Q1, Q3)]	73 (62, 80)	73 (60, 80)	0.6	
Male sex	132 (66%)	408 (65%)	0.8	
**Outcomes**				
30-day survival	20 (10%)	119 (19%)	0.003	
6-month survival	17 (8.7%)	105 (17%)	0.004	
Functional status at discharge			0.035	
CPC 1			17 (94%)	83 (75%)
CPC 2	0 (0%)	24 (22%)		
CPC 3	1 (5.6%)	3 (2.7%)		
**Reason for admission**				
Acute myocardial infarction	41 (21%)	88 (14%)	0.029	
Chronic kidney disease	7 (3.5%)	28 (4.5%)	0.6	
Cancer	10 (5.0%)	33 (5.3%)	0.9	
Heart failure or acute pulmonary edema	22 (11%)	84 (13%)	0.4	
Surgery or trauma	15 (7.5%)	34 (5.4%)	0.3	
Pneumonia or lung pathology	37 (19%)	99 (16%)	0.4	
**Direct cause**				
Acute respiratory distress syndrome	69 (36%)	208 (36%)	>0.9	
Arrhythmia	34 (18%)	126 (22%)	0.3	
Hypotension	58 (31%)	174 (30%)	0.9	
**Location**				
Operating room	6 (3.0%)	25 (4.0%)	0.5	
Ward	77 (39%)	205 (33%)	0.14	
Intensive care unit or high dependency unit	18 (9.0%)	64 (10%)	0.6	
Emergency department	32 (16%)	100 (16%)	>0.9	
**Pre-existing advanced life support procedures**				
Monitoring and intravenous catheter	99 (50%)	331 (53%)	0.4	
Mechanical ventilation	157 (82%)	493 (82%)	>0.9	
**Initial rhythm**				
Asystole	78 (39%)	230 (37%)	0.6	
Pulseless electrical activity	77 (39%)	245 (39%)	0.9	
Ventricular fibrillation	34 (17%)	127 (20%)	0.3	
Ventricular tachycardia	11 (5.5%)	24 (3.8%)	0.3	
**Resuscitation parameters**				
Cardiopulmonary resuscitation duration (min) [median (Q1, Q3)]	20 (6, 30)	20 (6, 30)	0.6	
Time to airway establishment (min) [median (Q1, Q3)]	3.00 (2.00, 4.00)	3.00 (2.00, 4.00)	0.7	
Cardiopulmonary resuscitation performance by the department staff or resuscitation team			0.6	
Department staff	19 (9.5%)	59 (9.4%)		
Advanced department staff	15 (7.5%)	62 (9.9%)		
Resuscitation team with or without department staff	166 (83%)	505 (81%)		
**Physicians and nurses**				
Physician present during resuscitation	54 (27%)	234 (37%)	0.007	
Nurse participation	178 (99%)	523 (96%)	0.018	
Nurse starting cardiopulmonary resuscitation	45 (25%)	125 (22%)	0.5	
**Resuscitation team**				
Time to resuscitation team call (min)			0.5	
0	115 (76%)	340 (77%)		
1	33 (22%)	95 (22%)		
2	3 (2.0%)	4 (0.9%)		
Time to resuscitation team arrival (min) [median (Q1, Q3)]	2.00 (2.00, 3.00)	2.00 (2.00, 3.00)	0.3	
**Witnesses/bystanders**				
Any witnesses/bystanders	190 (95%)	605 (97%)	0.3	
Physician or nurse	138 (69%)	459 (73%)	0.2	

Data are n (%) unless otherwise stated. Percentages may not sum to 100% due to rounding and/or missing data; denominators vary by variable. CPC is reported among patients discharged alive with available CPC documentation (weekend n = 18; weekday n = 110).

**Table 3:** Comparisons of patient-, event-, and system-level characteristics between morning and afternoon/night in-hospital cardiac arrests.

Variable	Morning (n = 321)	Afternoon/Night (n = 505)	*p*-Value
**Demographics**			
Age [median (Q1, Q3)]	73 (63, 80)	72 (60, 79)	0.4
Male sex	216 (67%)	324 (64%)	0.4
**Outcomes**			
30-day survival	70 (22%)	69 (14%)	0.002
6-month survival	63 (20%)	59 (12%)	0.001
Functional status at discharge			0.2
CPC 1	52 (78%)	48 (79%)	
CPC 2	11 (16%)	13 (21%)	
CPC 3	4 (6%)	0 (0%)	
**Reason for admission**			
Acute myocardial infarction	46 (14%)	83 (16%)	0.4
Chronic kidney disease	20 (6.2%)	15 (3.0%)	0.023
Cancer	23 (7.2%)	20 (4.0%)	0.043
Heart failure or acute pulmonary edema	48 (15%)	88 (17%)	0.4
Surgery or trauma	26 (8.1%)	23 (4.6%)	0.036
Pneumonia or lung pathology	50 (16%)	86 (17%)	0.6
**Direct cause**			
Acute respiratory distress syndrome	107 (35%)	170 (36%)	0.8
Arrhythmia	71 (23%)	89 (19%)	0.14
Hypotension	89 (29%)	143 (31%)	0.7
**Location**			
Operating room	23 (7.2%)	8 (1.6%)	<0.001
Ward	114 (36%)	168 (33%)	0.5
Intensive care unit or high dependency unit	30 (9.3%)	52 (10%)	0.7
Emergency department	44 (14%)	88 (17%)	0.2
**Pre-existing advanced life support procedures**			
Monitoring and intravenous catheter	170 (53%)	260 (51%)	0.7
Mechanical ventilation	262 (84%)	388 (80%)	0.2
**Initial rhythm**			
Asystole	111 (35%)	197 (39%)	0.2
Pulseless electrical activity	124 (39%)	198 (39%)	0.9
Ventricular fibrillation	74 (23%)	87 (17%)	0.039
Ventricular tachycardia	12 (3.7%)	23 (4.6%)	0.6
**Resuscitation parameters**			
Cardiopulmonary resuscitation duration (min) [median (Q1, Q3)]	15 (5, 30)	20 (10, 30)	0.043
Time to airway establishment (min) [median (Q1, Q3)]	3.00 (2.00, 4.00)	3.00 (2.00, 4.00)	0.04
Cardiopulmonary resuscitation performance by the department staff or resuscitation team			0.039
Department staff	22 (6.9%)	56 (11%)	
Advanced department staff	37 (12%)	40 (7.9%)	
Resuscitation team with or without department staff	262 (82%)	409 (81%)	
**Physicians and nurses**			
Physician present during resuscitation	124 (39%)	164 (32%)	0.07
Nurse participation	262 (96%)	439 (97%)	0.2
Nurse starting cardiopulmonary resuscitation	57 (20%)	113 (25%)	0.1
**Resuscitation team**			
Time to resuscitation team call (min)			>0.9
0	175 (78%)	280 (77%)	
1	48 (21%)	80 (22%)	
2	2 (0.9%)	5 (1.4%)	
Time to resuscitation team arrival (min) [median (Q1, Q3)]	2.00 (2.00, 3.00)	3.00 (2.00, 3.00)	0.037
**Witnesses/bystanders**			
Any witnesses/bystanders	313 (98%)	482 (95%)	0.13
Physician or nurse	237 (74%)	360 (71%)	0.4

Data are n (%) unless otherwise stated. Percentages may not sum to 100% due to rounding and/or missing data; denominators vary by variable. CPC is reported among patients discharged alive with available CPC documentation (morning n = 67; afternoon/night n = 61).

## Data Availability

The data presented in this study are available on request from the corresponding author.

## Supplementary Materials

The following supporting information can be downloaded at: https://www.mdpi.com/article/10.3390/jcm15030987/s1, Table S1: Complete comparisons of patient-, event-, and system-level characteristics between weekday and weekend in-hospital cardiac arrests, Table S2: Complete comparisons of patient-, event-, and system-level characteristics between morning and afternoon/night in-hospital cardiac arrests.

## Abbreviations

The following abbreviations are used in this manuscript:

aORs	adjusted Odds Ratios
CPC	Cerebral Performance Category
CAD	Coronary Artery Disease
CKD	Chronic Kidney Disease
DM	Diabetes Mellitus
DNAR	Do-Not-Attempt-Resuscitation
EU GDPR	European Union General Data Protection Regulation
HF	Heart Failure
HTN	Hypertension
ICU	Intensive Care Unit
IHCA	In-hospital Cardiac Arrest
OR	Operating Room
ROSC	Return Of Spontaneous Circulation
VHD	Valvular Heart Disease
VIFs	Variance Inflation Factors
