# Supplementary material for: Disparities in Survival After In-Hospital Cardiac Arrest by Time of Day and Day of Week: A Single-Center Cohort Study
Source: J Clin Med. 2026 Jan 26;15(3):987. doi: 10.3390/jcm15030987 (PMC12897753; doi:10.3390/jcm15030987)
Supplement: Supplementary file 1 [file jcm-15-00987-s001.zip › jcm-4084593-SI.pdf]

Supplementary Materials

Table S1. Complete comparisons of patient-, event-, and system-level characteristics between weekday and weekend in-hospital cardiac arrests.

Variable	Weekend (n=200)	Weekday (n=626)	p-value
**Demographics**			
Age [median (Q1, Q3)]	73 (62, 80)	73 (60, 80)	0.6
Male sex	132 (66%)	408 (65%)	0.8
**Outcomes**			
30-day survival	20 (10%)	119 (19%)	0.003
6-month survival	17 (8.7%)	105 (17%)	0.004
Functional status at discharge			0.035
CPC 1	17 (94%)	83 (75%)	
CPC 2	0 (0%)	24 (22%)	
CPC 3	1 (5.6%)	3 (2.7%)	
**Reason for admission**			
Acute myocardial infarction	41 (21%)	88 (14%)	0.029
Coronary artery disease	50 (25%)	127 (20%)	0.2
Chronic kidney disease	7 (3.5%)	28 (4.5%)	0.6
Cancer	10 (5.0%)	33 (5.3%)	0.9
Cardiac arrhythmia	9 (4.5%)	22 (3.5%)	0.5
Coma	3 (1.5%)	18 (2.9%)	0.3
Aortic dissection	4 (2.0%)	18 (2.9%)	0.5

Gastrointestinal pathology	13 (6.5%)	36 (5.8%)	0.7
Heart failure or acute pulmonary edema	22 (11%)	84 (13%)	0.4
Hematological pathology	4 (2.0%)	12 (1.9%)	>0.9
Stroke or central nervous system pathology	11 (5.5%)	27 (4.3%)	0.5
Surgery or trauma	15 (7.5%)	34 (5.4%)	0.3
Syncope	4 (2.0%)	11 (1.8%)	0.8
Valvular heart disease or endocarditis	14 (7.0%)	70 (11%)	0.088
Pneumonia or lung pathology	37 (19%)	99 (16%)	0.4
**Comorbidities**			
Coronary artery disease	37 (19%)	119 (19%)	0.9
Chronic kidney disease	9 (4.5%)	33 (5.3%)	0.7
Cancer	17 (8.5%)	40 (6.4%)	0.3
Diabetes mellitus	4 (2.0%)	6 (1.0%)	0.3
Heart failure	33 (17%)	103 (16%)	>0.9
Hypertension	4 (2.0%)	17 (2.7%)	0.6
Mental disease	2 (1.0%)	13 (2.1%)	0.5
Lung pathology	9 (4.5%)	26 (4.2%)	0.8
Valvular heart disease	5 (2.5%)	16 (2.6%)	>0.9
Stroke or central nervous system pathology	7 (3.5%)	16 (2.6%)	0.5
**Direct cause**			
Acute respiratory distress syndrome	69 (36%)	208 (36%)	>0.9
Arrhythmia	34 (18%)	126 (22%)	0.3
Hypotension	58 (31%)	174 (30%)	0.9

Sepsis	4 (2.1%)	9 (1.6%)	0.5
Myocardial infarction	25 (13%)	63 (11%)	0.4
**Location**			
Operating room	6 (3.0%)	25 (4.0%)	0.5
Dialysis unit	8 (4.0%)	26 (4.2%)	>0.9
Catheterization laboratory	11 (5.5%)	39 (6.2%)	0.7
Ward	77 (39%)	205 (33%)	0.14
Intensive care unit or high dependency unit	18 (9.0%)	64 (10%)	0.6
Emergency department	32 (16%)	100 (16%)	>0.9
Coronary care unit	48 (24%)	167 (27%)	0.5
Advanced department	15 (7.5%)	60 (9.6%)	0.4
**Timing**			
Shift			0.058
Morning	78 (39%)	243 (39%)	
Afternoon	55 (28%)	127 (20%)	
Night	67 (34%)	256 (41%)	
**Pre-existing advanced life support procedures**			
None present	10 (5.0%)	27 (4.3%)	0.7
Intravenous catheter	190 (95%)	599 (96%)	0.7
Monitoring and intravenous catheter	99 (50%)	331 (53%)	0.4
Laryngeal mask airway or bag valve mask	24 (13%)	85 (14%)	0.6
Mechanical ventilation	157 (82%)	493 (82%)	>0.9
All present	17 (8.5%)	73 (12%)	0.2

**Initial rhythm**			
Asystole	78 (39%)	230 (37%)	0.6
Pulseless electrical activity	77 (39%)	245 (39%)	0.9
Ventricular fibrillation	34 (17%)	127 (20%)	0.3
Ventricular tachycardia	11 (5.5%)	24 (3.8%)	0.3
**Resuscitation parameters**			
Cardiopulmonary resuscitation duration (min) [median (Q1, Q3)]	20 (6, 30)	20 (6, 30)	0.6
Chest compressions	68 (77%)	207 (70%)	0.2
Ventilation	26 (48%)	79 (43%)	0.5
Number of delivered shocks			0.12
0	10 (22%)	41 (27%)	
1	11 (24%)	59 (39%)	
2	13 (29%)	26 (17%)	
3	11 (24%)	26 (17%)	
Total adrenaline dose (ampules) [median (Q1, Q3)]	4.00 (2.00, 6.00)	4.00 (2.00, 6.00)	0.2
Total amiodarone dose (milligrams)			0.4
150	0 (0%)	3 (6.8%)	
300	14 (93%)	40 (91%)	
450	1 (6.7%)	1 (2.3%)	
No ventilation needed	6 (3.1%)	13 (2.2%)	0.4
Tracheostomy	5 (2.6%)	13 (2.2%)	0.8

Time to first adrenaline dose (min) [median (Q1, Q3)]	2.00 (1.00, 3.00)	2.00 (1.00, 3.00)	0.13
Time to airway establishment (min) [median (Q1, Q3)]	3.00 (2.00, 4.00)	3.00 (2.00, 4.00)	0.7
Cardiopulmonary resuscitation performance by the department staff or resuscitation team			0.6
Department staff	19 (9.5%)	59 (9.4%)	
Advanced department staff	15 (7.5%)	62 (9.9%)	
Resuscitation team with or without department staff	166 (83%)	505 (81%)	
**Physicians and nurses**			
Physician present during resuscitation	54 (27%)	234 (37%)	0.007
Nurse participation	178 (99%)	523 (96%)	0.018
Nurse starting cardiopulmonary resuscitation	45 (25%)	125 (22%)	0.5
**Resuscitation team**			
Resuscitation team call	150 (75%)	439 (70%)	0.2
Time to resuscitation team call (min)			0.5
0	115 (76%)	340 (77%)	
1	33 (22%)	95 (22%)	
2	3 (2.0%)	4 (0.9%)	
Time to resuscitation team arrival (min) [median (Q1, Q3)]	2.00 (2.00, 3.00)	2.00 (2.00, 3.00)	0.3
Resuscitation team present	16 (8.0%)	66 (11%)	0.3
Crash cart present	139 (98%)	427 (94%)	0.094
**Witnesses/bystanders**			

Any witnesses/bystanders	190 (95%)	605 (97%)	0.3
Physician or nurse	138 (69%)	459 (73%)	0.2
Resuscitation team	12 (6.0%)	53 (8.5%)	0.3
Accompanying person	40 (20%)	93 (15%)	0.085
**Other**			
Previous arrest	3 (1.7%)	3 (0.6%)	0.2

* Data are n (%) unless otherwise stated. Percentages may not sum to 100% due to rounding and/or missing data; denominators vary by variable. CPC is reported among patients discharged alive with available CPC documentation (Weekend n=18; Weekday n=110).

Table S2. Complete comparisons of patient-, event-, and system-level characteristics between morning and afternoon/night in-hospital cardiac arrests.

Variable	Morning (n=321)	Afternoon/night (n=505)	p-value
**Demographics**			
Age [median (Q1, Q3)]	73 (63, 80)	72 (60, 79)	0.4
Male sex	216 (67%)	324 (64%)	0.4
**Outcomes**			
30-day survival	70 (22%)	69 (14%)	0.002
6-month survival	63 (20%)	59 (12%)	0.001
Functional status at discharge			0.2
CPC 1	52 (78%)	48 (79%)	
CPC 2	11 (16%)	13 (21%)	
CPC 3	4 (6%)	0 (0%)	

**Reason for admission**			
Acute myocardial infarction	46 (14%)	83 (16%)	0.4
Coronary artery disease	63 (20%)	114 (23%)	0.3
Chronic kidney disease	20 (6.2%)	15 (3.0%)	0.023
Cancer	23 (7.2%)	20 (4.0%)	0.043
Cardiac arrhythmia	11 (3.4%)	20 (4.0%)	0.7
Coma	8 (2.5%)	13 (2.6%)	>0.9
Aortic dissection	5 (1.6%)	17 (3.4%)	0.12
Gastrointestinal pathology	13 (4.0%)	36 (7.1%)	0.068
Heart failure or acute pulmonary edema	48 (15%)	88 (17%)	0.4
Hematological pathology	5 (1.6%)	11 (2.2%)	0.5
Stroke or central nervous system pathology	13 (4.0%)	25 (5.0%)	0.5
Surgery or trauma	26 (8.1%)	23 (4.6%)	0.036
Syncope	6 (1.9%)	9 (1.8%)	>0.9
Valvular heart disease or endocarditis	33 (10%)	51 (10%)	>0.9
Pneumonia or lung pathology	50 (16%)	86 (17%)	0.6
**Comorbidities**			
Coronary artery disease	67 (21%)	89 (18%)	0.2
Chronic kidney disease	18 (5.6%)	24 (4.8%)	0.6
Cancer	23 (7.2%)	34 (6.7%)	0.8
Diabetes mellitus	4 (1.2%)	6 (1.2%)	>0.9
Heart failure	48 (15%)	88 (17%)	0.4
Hypertension	8 (2.5%)	13 (2.6%)	>0.9

Mental disease	7 (2.2%)	8 (1.6%)	0.5
Lung pathology	15 (4.7%)	20 (4.0%)	0.6
Valvular heart disease	10 (3.1%)	11 (2.2%)	0.4
Stroke or central nervous system pathology	10 (3.1%)	13 (2.6%)	0.6
**Direct cause**			
Acute respiratory distress syndrome	107 (35%)	170 (36%)	0.8
Arrhythmia	71 (23%)	89 (19%)	0.14
Hypotension	89 (29%)	143 (31%)	0.7
Sepsis	3 (1.0%)	10 (2.1%)	0.2
Myocardial infarction	33 (11%)	55 (12%)	0.7
**Location**			
Operating room	23 (7.2%)	8 (1.6%)	<0.001
Dialysis unit	14 (4.4%)	20 (4.0%)	0.8
Catheterization laboratory	20 (6.2%)	30 (5.9%)	0.9
Ward	114 (36%)	168 (33%)	0.5
Intensive care unit or high dependency unit	30 (9.3%)	52 (10%)	0.7
Emergency department	44 (14%)	88 (17%)	0.2
Coronary care unit	76 (24%)	139 (28%)	0.2
Advanced department	37 (12%)	38 (7.5%)	0.051
**Timing**			
Weekend	78 (24%)	122 (24%)	>0.9
**Pre-existing advanced life support procedures**			

None present	14 (4.4%)	23 (4.6%)	0.9
Intravenous catheter	307 (96%)	482 (95%)	0.9
Monitoring and intravenous catheter	170 (53%)	260 (51%)	0.7
Laryngeal mask airway or bag valve mask	38 (12%)	71 (15%)	0.3
Mechanical ventilation	262 (84%)	388 (80%)	0.2
All present	35 (11%)	55 (11%)	>0.9
**Initial rhythm**			
Asystole	111 (35%)	197 (39%)	0.2
Pulseless electrical activity	124 (39%)	198 (39%)	0.9
Ventricular fibrillation	74 (23%)	87 (17%)	0.039
Ventricular tachycardia	12 (3.7%)	23 (4.6%)	0.6
**Resuscitation parameters**			
Cardiopulmonary resuscitation duration (min) [median (Q1, Q3)]	15 (5, 30)	20 (10, 30)	0.043
Chest compressions	91 (63%)	184 (77%)	0.002
Ventilation	31 (33%)	74 (51%)	0.004
Total adrenaline dose (ampules) [median (Q1, Q3)]	3.00 (2.00, 6.00)	4.00 (2.00, 6.00)	0.2
Total amiodarone dose (milligrams)			0.3
150	0 (0%)	3 (8.1%)	
300	22 (100%)	32 (86%)	
450	0 (0%)	2 (5.4%)	
No ventilation needed	5 (1.6%)	14 (2.9%)	0.2
Tracheostomy	7 (2.2%)	11 (2.3%)	>0.9

Time to first adrenaline dose (min) [median (Q1, Q3)]	2.00 (1.00, 3.00)	2.00 (1.00, 3.00)	0.12
Time to first defibrillation (min)			0.6
0	25 (29%)	26 (24%)	
1	31 (36%)	39 (35%)	
2	18 (21%)	21 (19%)	
3	13 (15%)	24 (22%)	
Time to airway establishment (min) [median (Q1, Q3)]	3.00 (2.00, 4.00)	3.00 (2.00, 4.00)	0.04
Cardiopulmonary resuscitation performance by the department staff or resuscitation team			0.039
Department staff	22 (6.9%)	56 (11%)	
Advanced department staff	37 (12%)	40 (7.9%)	
Resuscitation team with or without department staff	262 (82%)	409 (81%)	
**Physicians and nurses**			
Physician present during resuscitation	124 (39%)	164 (32%)	0.07
Nurse participation	262 (96%)	439 (97%)	0.2
Nurse starting cardiopulmonary resuscitation	57 (20%)	113 (25%)	0.1
**Resuscitation team**			
Resuscitation team call	225 (70%)	364 (72%)	0.5
Time to resuscitation team call (min)			>0.9
0	175 (78%)	280 (77%)	
1	48 (21%)	80 (22%)	

2	2 (0.9%)	5 (1.4%)	
Time to resuscitation team arrival (min) [median (Q1, Q3)]	2.00 (2.00, 3.00)	3.00 (2.00, 3.00)	0.037
Resuscitation team present during arrest	37 (12%)	45 (8.9%)	0.2
Arrest after resuscitation team arrival	37 (12%)	48 (9.5%)	0.4
Crash cart present	207 (95%)	359 (95%)	>0.9
**Witnesses/bystanders**			
Any witnesses/bystanders	313 (98%)	482 (95%)	0.13
Physician or nurse	237 (74%)	360 (71%)	0.4
Resuscitation team	27 (8.4%)	38 (7.5%)	0.6
Accompanying person	49 (15%)	84 (17%)	0.6
**Other**			
Previous arrest	4 (1.4%)	2 (0.5%)	0.2

* Data are n (%) unless otherwise stated. Percentages may not sum to 100% due to rounding and/or missing data; denominators vary by variable. CPC is reported among patients discharged alive with available CPC documentation (Morning n=67; Afternoon/night n=61).
